# Self-reported HIV testing and treatment among migrants from northeast South Africa: A cross-sectional, population-based analysis

**DOI:** 10.4102/sajhivmed.v26i1.1666

**Published:** 2025-04-30

**Authors:** Rachel R. Yorlets, F. Xavier Goméz-Olivé, Carren Ginsburg, Sadson Harawa, Kathleen Kahn, Stephen Tollman, Mark Collinson, Mark Lurie

**Affiliations:** 1 Division of Infectious Diseases, Boston Medical Center, Boston, United States; 2 South African Medical Research Council/Wits Rural Public Health and Health Transitions Research Unit (Agincourt), School of Public Health, Faculty of Health Sciences, University of the Witwatersrand, Johannesburg, South Africa; 3 Department of Epidemiology, Brown University School of Public Health, Providence, Rhode Island, United States

## Introduction

Adults who move temporarily within South Africa from rural homes to urban economic and educational opportunities are highly prevalent and historically well documented as high-risk^1,2,3,4^ within the country’s generalised HIV epidemic, but less is known about these temporary internal migrants’ HIV care engagement in today’s universal ‘test and treat’ (UTT) era.^5^ Before persons living with HIV (PLWH) were eligible to start antiretroviral therapy (ART) immediately at diagnosis, researchers found that migrants were less likely to test for HIV,^6^ be diagnosed, and to both start and stay in care compared with their non-migratory counterparts.^7,8,9,10,11^ The UTT policy may be especially beneficial for migrants, but it is unclear if or how it has changed migrants’ HIV care interactions, as the health system remains unresponsive to migrant-specific needs.^12^ Historically, mobile populations in South Africa have experienced significant disruption events,^13,14,15^ but in the last decade, scientists suggest within-country migration is becoming a relatively more selective and less stressful process,^16^ as circumstances surrounding moves have become less dire.

South Africa has reached 95-79-91^17^ on the United Nations’ global 95-95-95 targets to end HIV by 2030: 95% of PLWH know their status, 95% of which adhere to treatment, and 95% of which achieve viral suppression,^18^ but we have no estimates of migrants’ progression through HIV care. Expansions in HIV testing suggest that we may not observe a difference in testing and receiving results by migration status but, given migrants’ heightened HIV acquisition risk and barriers to care continuity, we suspect that migrants are more likely to self-report a positive HIV test and less likely to report ART adherence. We used UTT era data from one of the country’s health and sociodemographic surveillance systems to describe migrants’ self-reported history of HIV testing, diagnosis, and treatment. We also synthesised the sociodemographic profile of migrants who were engaged at each of these three stages of care. With the knowledge that migrants in South Africa have been historically vulnerable within the HIV epidemic, we aimed to contribute to understanding the migrant experience across the HIV care continuum in the modern UTT era.

## Research methods and design

### Study setting

Since 1992, the South African Medical Research Council (SAMRC)/Wits Rural Public Health and Health Transitions Research Unit’s Agincourt Health and Sociodemographic Surveillance System has conducted a yearly census within its 420 square-kilometre study site in the Agincourt area of Mpumalanga province. The census records all births, deaths, and in- and out-migrations in some 20 000 households in 31 contiguous rural villages. Within the population (*N* > 116 000)^19^, there is a high prevalence of HIV and internal migration,^20^ making this an ideal setting for collecting data on self-reported HIV testing and treatment by migration status. We used data from the Migration Health and Follow-Up Study (MHFUS),^21^ which is a simple random sample of the 2016 Agincourt HDSS census; MHFUS included 3800 Xitsonga-speaking African participants aged 18–40 years, including migrants and permanent Agincourt residents. From February 2018 into early 2019, 3103 (82% of the original sample of 3800 MHFUS participants) respondents completed the MHFUS Wave 1 interview, which we used for these analyses.

### Migration status

Participants were asked for their current usual residence, where they ‘typically spent four or more nights a week over the past year’. If respondents identified one of the 31 villages within the Agincourt study site, they were categorised as residents; otherwise, they were migrants. The open-ended residence question was validated through branched questions asking the province and village of current residence; both methods yielded nearly identical categorisations.

### Sociodemographic characteristics

Participants confirmed their date of birth and sex from the Agincourt HDSS survey and reported their education, employment, and individual-level income for the prior month.

### HIV care

At the end of the interview, participants who reported a history of an HIV test when asked and reported that they had received their results were asked, ‘If you wouldn’t mind sharing, what was the result of your most recent HIV test?’ Participants were asked about ART history.

### Analysis

We generated descriptive statistics to quantify migrants’ and non-migrants’ self-reported HIV testing, status, and treatment history. We created a sociodemographic profile to characterise individuals who were retained at each stage.

### Ethical considerations

Ethical clearance to conduct this study was obtained from the University of the Witwatersrand Human Research Ethics Committee (Medical) (reference no.: M170277) and Institutional Review Board Authorization Agreement #17–46 with Brown University (reference no.: IRB00001223).

## Results

**FIGURE 1:**
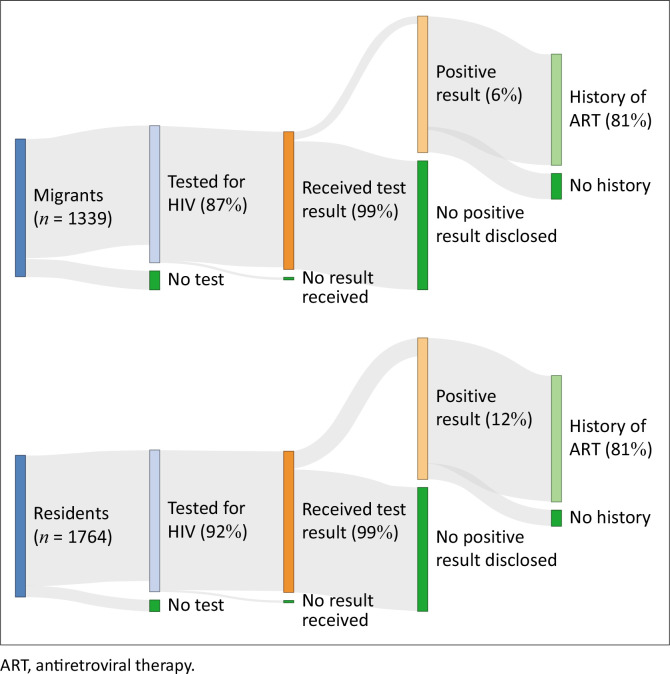
Self-reported history of HIV testing, status, and antiretroviral therapy uptake among migrants and residents (*N* = 3103).

Among participants who completed the baseline questionnaire (*N* = 3103), 57% (*n* = 1764) were Agincourt residents, and 43% (*n* = 1339) were migrants who had been living away from their rural home for four or more nights a week over the prior year. A higher proportion of residents than migrants reported a history of an HIV test (92% vs 87%), diagnosis (12% vs 6%), and ART (89% vs 81%) (Figure 1).

The MHFUS was age-restricted to adults 18–40 years old; in our sample, migrants and residents were in their late-twenties, on average. Compared to residents, a higher proportion of migrants were men, employed, had a secondary education, and earning an income (Table 1). Sociodemographic characteristics of migrants and residents who reported a prior HIV test did not differ from participants overall. For example, about two-thirds of all migrants and half of all residents reported a secondary or higher level of education; the same percentage of migrants and residents who reported a prior HIV test had a secondary education.

**TABLE 1:** Self-reported sociodemographic characteristics of participants who have received phases of HIV care.

Characteristics	Baseline		History of HIV test		Received HIV test results		HIV-positive result		History of ART	
	Migrants (*n* = 1339)	Residents (*n* = 1764)	Migrants (*n* = 1162)	Residents (*n* = 1619)	Migrants (*n* = 1149)	Residents (*n* = 1609)	Migrants (*n* = 74)	Residents (*n* = 198)	Migrants (*n* = 60)	Residents (*n* = 177)
Age in years (mean ± s.d.)	29.09 ± 5.33	27.76 ± 6.04	29.21 ± 5.23	27.95 ± 5.97	29.17 ± 5.22	27.94 ± 5.97	32.47 ± 5.13	31.76 ± 5.13	32.82 ± 4.71	31.89 ± 5.14
**Sex, *n* (%)**										
Women	553 (41.3)	988 (56.0)	503 (43.3)	968 (59.8)	500 (43.5)	963 (59.9)	56 (75.7)	159 (80.3)	46 (76.7)	144 (81.4)
Men	786 (58.7)	776 (44.0)	659 (56.7)	651 (40.2)	649 (56.5)	646 (40.1)	18 (24.3)	39 (19.7)	14 (23.3)	33 (18.6)
**Education, *n* (%)**										
Elementary or less	414 (30.9)	914 (51.8)	363 (31.2)	810 (50.0)	361 (31.4)	803 (49.9)	41 (55.4)	138 (69.7)	37 (61.7)	128 (72.3)
Secondary or more	925 (69.1)	850 (48.2)	799 (68.8)	809 (50.0)	788 (68.6)	806 (50.1)	33 (44.6)	60 (30.3)	23 (38.3)	49 (27.7)
**Employment, *n* (%)**										
Unemployed	445 (33.2)	1231 (69.8)	373 (32.1)	1121 (69.2)	370 (32.2)	1115 (69.3)	24 (32.4)	136 (68.7)	20 (33.3)	120 (67.8)
Employed	894 (66.8)	533 (30.2)	789 (67.9)	498 (30.8)	779 (67.8)	494 (30.7)	50 (67.6)	62 (31.3)	40 (66.7)	57 (32.2)
**Income, prior month, *n* (%)**										
ZAR 0	478 (35.7)	1219 (69.1)	401 (34.5)	1112 (68.7)	398 (34.6)	1106 (68.7)	25 (33.8)	132 (66.7)	21 (35.0)	116 (65.5)
ZAR 1 – ZAR 6400	497 (37.1)	441 (25.0)	436 (37.5)	411 (25.4)	434 (37.8)	407 (25.3)	44 (59.5)	61 (30.8)	36 (60.0)	56 (31.6)
> ZAR 6400	364 (27.2)	104 (5.9)	325 (28.0)	96 (5.9)	317 (27.6)	96 (6.0)	5 (6.8)	5 (2.5)	3 (5.0)	5 (2.8)

ART, antiretroviral therapy; s.d., standard deviation; ZAR, South African Rand.

Regardless of migration status, participants who reported living with HIV, and those who reported prior ART use, were disproportionately women, without a secondary education, and, on average, 3–4 years older than the mean age of all participants who received test results.

## Conclusion

In our cross-sectional analysis in South Africa’s UTT era, Agincourt residents were more likely than migrants with ties to Agincourt to report a history of HIV testing, diagnosis, and treatment, consistent with migrants’ historic challenges to healthcare engagement while living away from home. Our sample also reflected demographic norms of migrants as men who move for work: migrant participants were mostly men, employed, and had at least a secondary education. Since our sample was predominantly male and previous Agincourt estimates found that up to 60% of its men migrate,^22^ it was unexpected that most of our participants (57%) were non-migrants, perhaps suggesting that migrants were under-represented in our sample.

We had suspected that, in the UTT era, HIV testing would not differ by migration status, but a higher proportion of residents reported a prior test (92% vs 87%). Sociodemographic features were similar among residents and migrants who had been tested. While HIV testing has expanded through home-based and clinic-based tests and uptake is relatively high,^23^ our finding that non-migrants were more likely to have tested for HIV is consistent with evidence that residents in this cohort are more likely to use healthcare.^21^

While we thought migrants would be more likely to report an HIV diagnosis, we found the opposite: 12% of residents who had tested reported a positive status versus 6% of migrants. This was surprising given migrants’ heightened risk of HIV acquisition,^1,2,3,4^ and our prior finding that the sensitivity of self-reported HIV-positive status was similar between MHFUS Wave 1 residents and migrants who consented to a study-administered dried blood spot HIV test – a subset (*n* = 1918) of the analytic sample used here (*N* = 3103).^24^ Without the ability to verify HIV status among all participants, we cannot know if our findings reflect a truly lower prevalence of migrants living with HIV at study baseline.

As suspected, migrants who disclosed a positive status were less likely than their resident counterparts to report a history of ART use (81% vs 89%). Other studies have reported that migrants living with HIV are less likely to start and stay in HIV care.^7,8,9,10,11,25,26^ Some explanations for migrants’ reduced ART adherence^7^ point to lifestyle disruption events inherent to mobility, including changes to healthcare,^13,14,15^ and navigating a health system designed for stable populations; migrants often have a different primary language^7^ and cultural norms.

Regardless of migration status, participants who reported a positive test and a history of ART were 3–4 years older, on average, disproportionately women, and less educated than those who reported a negative test or no treatment history. Women may be predominant because of their access to HIV testing during antenatal care, the ‘missing men’ phenomenon, or a truly higher prevalence among women. We may observe a lower education level among persons who disclosed their diagnosis and treatment if disclosure is perceived to be less risky.

We are limited by our inability to verify self-reported data, particularly participants’ history of HIV diagnosis or treatment, which may have been misclassified as a result of social desirability. In a previous analysis within this cohort, Yorlets et al. found that self-reported HIV diagnosis was highly predictive and insensitive,^24^ providing further evidence that laboratory test results are needed to validate self-reported negative status. Finally, while this is a cross-sectional analysis, temporality of migration status is established by the questionnaire, which asks about residence in the prior year.

We provide evidence in the UTT era that internal migrants in South Africa are less likely to start and stay in HIV care compared to their non-migrant counterparts, which supports historic evidence. Routine surveillance of self-reported HIV care engagement is needed to supplement laboratory-based results to evaluate mismatches between what PLWH need and what they receive. Alongside the global recognition that migration is a determinant of health,^27^ our findings support existing calls for migrant-specific policies to increase migrants’ HIV care uptake.
